# Association of Serum *P*-Cresyl Sulfate Level with Peripheral Artery Disease in Kidney Transplantation Patients

**DOI:** 10.3390/jcm15093302

**Published:** 2026-04-26

**Authors:** Hsiao-Hui Yang, Yen-Cheng Chen, Chin-Hung Liu, Bang-Gee Hsu

**Affiliations:** 1 Division of General Surgery, Department of Surgery, Hualien Tzu Chi Hospital, Buddhist Tzu Chi Medical Foundation, Hualien 97004, Taiwan; 2 School of Medicine, Tzu Chi University, Hualien 97004, Taiwan; 3 Institute of Medical Sciences, Tzu Chi University, Hualien 97004, Taiwan; 4 Graduate Institute of Clinical Pharmacy, School of Medicine, Tzu Chi University, Hualien 97004, Taiwan; 5 School of Pharmacy, Tzu Chi University, Hualien 97004, Taiwan; 6 Division of Nephrology, Hualien Tzu Chi Hospital, Buddhist Tzu Chi Medical Foundation, Hualien 97004, Taiwan

**Keywords:** ankle-brachial index, kidney transplantation, *p*-cresyl sulfate, peripheral artery disease

## Abstract

**Background**: *p*-Cresyl sulfate (PCS) has been linked to vascular dysfunction through endothelial injury and vascular remodeling. Peripheral artery disease (PAD), identified by a low ankle–brachial index (ABI), is associated with increased mortality in kidney transplant (KT) recipients. This study investigated the association between serum PCS levels and PAD (as defined by ABI) in KT recipients. **Methods**: This cross-sectional, single-center study included 90 KT recipients. Serum total PCS levels were quantified using liquid chromatography–mass spectrometry. ABI was measured using an automated oscillometric device, and PAD was defined as ABI < 0.9. **Results**: Among the 90 KT recipients, 20 (22.2%) met the ABI for PAD. Patients with ABI-defined PAD had a significantly higher prevalence of diabetes mellitus (*p* = 0.036) and serum PCS levels (*p* = 0.001). Multivariate logistic regression analysis adjusting for potential confounders revealed that serum PCS levels remained independently associated with PAD (odds ratio 1.254, 95% confidence interval 1.108–1.419; *p* < 0.001). PCS levels were inversely correlated with both left (*r* = −0.339, *p* = 0.001) and right (*r* = −0.357, *p* < 0.001) ABIs. The association remained consistent in penalized regression models. **Conclusions**: Higher serum PCS levels were independently associated with ABI-defined PAD in KT recipients. The findings indicate that residual uremic toxin burden may contribute to peripheral vascular disease despite the restoration of renal function following transplantation.

## 1. Introduction

Cardiovascular complications continue to be a major contributor to morbidity and mortality among kidney transplant (KT) recipients. Peripheral vascular disease commonly develops among patients before and after KT and is further associated with impaired transplant outcomes [1]. A combination of traditional cardiovascular risk factors and transplant-related conditions predisposes this population to vascular injury, with disease progression driven by the overlapping processes of atherosclerosis and arteriosclerosis [1].

Peripheral artery disease (PAD) refers to atherosclerotic narrowing of the lower extremity arteries and is commonly defined by an ankle–brachial index (ABI) below 0.90. It affects more than 200 million people worldwide [2]. PAD shares major risk factors with coronary artery disease, including advanced age, hypertension, smoking, and diabetes mellitus, with the latter two appearing to exert particularly strong effects [3,4]. In KT recipients, PAD is associated with adverse outcomes. Severe lower extremity PAD has been reported in 4–6% of transplant recipients within 5–10 years of follow-up [5], with ABI-defined PAD being linked to increased mortality and graft failure [6,7]. These findings highlight the importance of early detection of PAD in this population.

Aside from traditional risk factors, protein-bound uremic toxins have been identified as important contributors to vascular injury in chronic kidney disease (CKD). In particular, *p*-cresyl sulfate (PCS) is produced by the intestinal bacterial fermentation of tyrosine, then undergoes hepatic sulfation before entering the circulation, where it is predominantly bound to albumin [8]. Its strong protein-binding properties render PCS resistant to glomerular clearance and conventional dialysis removal, resulting in progressive accumulation across advancing stages of CKD and its persistence even after transplantation [8,9].

Clinical evidence links elevated circulating PCS levels to cardiovascular events, mortality, and CKD progression [9,10,11,12,13,14]. Moreover, PCS levels remain elevated in KT recipients despite improved glomerular filtration [15], suggesting that the restoration of renal function alone may not fully mitigate toxin-related vascular injury. Data on the relationship between PCS and ABI-defined PAD in KT recipients remain limited. Given the clinical relevance of ABI-defined PAD in this population, we explored the association between circulating PCS levels and ABI-defined PAD.

## 2. Materials and Methods

### 2.1. Patients

This cross-sectional study enrolled 107 KT recipients at a medical center in Hualien, Taiwan, between 1 December 2021, and 30 June 2022. Written informed consent was obtained from all participants prior to enrollment. All participants had dialysis before transplantation. Data on baseline demographics, chronic medication use, dialysis duration before KT, and relevant medical histories were collected through structured interviews and medical record review. Information on immunosuppressive therapy, including tacrolimus, mycophenolate mofetil, corticosteroids, rapamycin, and cyclosporine, was extracted from medical records. Hypertension was defined by documented use of antihypertensive agents, and diabetes mellitus was established based on medical history or antidiabetic medication use. Exclusion criteria included refusal to participate (*n* = 3); the presence of malignancy (*n* = 3), acute infection (*n* = 1), acute rejection episode (*n* = 1), myocardial infarction (*n* = 1), stroke (*n* = 1), limb amputation (*n* = 3), or heart failure (*n* = 1); current treatment with cilostazol or pentoxifylline at the time of blood sampling (*n* = 2); and an ABI > 1.3 (*n* = 1). Finally, a total of 90 KT recipients were included in this study. The study was approved by the Research Ethics Committee of Hualien Tzu Chi Hospital, Buddhist Tzu Chi Medical Foundation (IRB108-219-A), and all procedures were performed in accordance with the Declaration of Helsinki.

### 2.2. Anthropometric and Biochemical Measurements

Body mass index was calculated as body weight (kg) divided by height squared (m^2^). Fasting blood samples were obtained and analyzed using an automated chemistry analyzer (Advia 1800; Siemens Healthcare GmbH, Erlangen, Germany) to determine glucose, glycated hemoglobin (HbA1c), lipid profile, renal function parameters, and electrolytes. Serum intact parathyroid hormone (iPTH) levels were measured using an enzyme-linked immunosorbent assay (Abcam, Cambridge, MA, USA) [16]. Estimated glomerular filtration rate (eGFR) was calculated using the CKD-EPI equation.

### 2.3. Measurement of Serum Total P-Cresyl Sulfate Levels

Serum total PCS concentrations were quantified using a high-performance liquid chromatography system (Waters e2695, Waters Corporation, Milford, MA, USA) coupled with a mass spectrometer (ACQUITY QDa; Waters Corporation, Milford, MA, USA), as previously described [17]. PCS was detected by mass spectrometry, and data were processed using Empower^®^ 3.0 software (Waters Corporation, New York, NY, USA).

### 2.4. ABI Measurements

Ankle–brachial index (ABI) was assessed using an oscillometric device (VaSera VS-1000; Fukuda Denshi, Tokyo, Japan) with participants in the supine position [16]. Blood pressure measurements were obtained at the brachial, dorsalis pedis, and posterior tibial arteries, and the highest ankle systolic pressure was divided by the highest brachial systolic pressure to calculate ABI for each limb. Continuous electrocardiographic monitoring was performed during the examination. Peripheral artery disease (PAD) was defined as an ABI < 0.9 in either limb according to established criteria [16].

### 2.5. Statistical Analysis

Statistical analyses were conducted using SPSS version 25.0 (IBM Corp., Armonk, NY, USA) and R software (version 4.2.2; R Foundation for Statistical Computing, Vienna, Austria). Data distribution was evaluated using the Kolmogorov–Smirnov test. Continuous variables are presented as mean ± standard deviation or median (interquartile range), as appropriate, and were compared using Student’s *t*-test or the Mann–Whitney U test (dialysis duration, TG, HDL-C, fasting glucose, HbA1c, BUN, creatinine, and iPTH). Categorical variables were analyzed using the χ^2^ test. Variables associated with ABI-defined PAD at *p* < 0.2 on univariate analysis were entered into multivariable logistic regression models. Variance inflation factors were calculated for all covariates to examine potential multicollinearity. Associations between PCS levels and clinical variables were examined using Spearman’s rank correlation coefficient. Because the number of ABI-defined PAD events was limited relative to the number of candidate predictors, penalized logistic regression approaches—including LASSO, ridge regression, and elastic net—were additionally applied as sensitivity analyses to reduce overfitting and improve estimate stability. All continuous predictors were standardized before analysis. Optimal tuning parameters were selected using k-fold cross-validation. The statistical significance of penalized regression coefficients was assessed using a bootstrap procedure with 1000 resamples. A *p*-value < 0.05 was considered statistically significant (two-sided).

## 3. Results

Table 1 summarizes the baseline clinical characteristics of the 90 KT recipients. Twenty patients (22.2%) met the diagnostic criteria for ABI-defined PAD, while the remaining 70 (77.8%) served as the normal ABI group. Compared with the normal ABI group, patients with ABI-defined PAD exhibited a significantly higher prevalence of diabetes mellitus (*p* = 0.036) and higher serum PCS levels (*p* = 0.001). However, the two groups did not differ significantly in age, sex, body mass index, blood pressure, lipid profiles, renal function parameters, donor type, dialysis duration, smoking, or the use of immunosuppressive and lipid-lowering medications.

Multivariate logistic regression analysis adjusting for variables with *p* < 0.2 in univariate comparisons (including diastolic blood pressure, TG, LDL-C, fasting glucose, creatinine, eGFR, iPTH, diabetes mellitus, and PCS levels) revealed that serum PCS concentration remained independently associated with ABI-defined PAD (odds ratio: 1.254, 95% confidence interval [CI]: 1.108–1.419; *p* < 0.001) (Table 2). All covariates had a variance inflation factor < 1.5, indicating no significant multicollinearity among the predictors.

To assess the robustness of the observed association, three penalized logistic regression models, LASSO, ridge regression, and elastic net, were applied. Serum PCS consistently demonstrated a significant positive association with ABI-defined PAD across all three models (all *p* < 0.001) (Table 3). In the penalized regression analyses, eGFR showed a positive association and fasting glucose showed an inverse association with ABI-defined PAD; however, these findings were not consistently supported by the conventional multivariable model (Table 2) or correlation analyses and should therefore be interpreted cautiously. Diabetes mellitus reached statistical significance in the ridge and elastic net models. Diastolic blood pressure, creatinine, TG, LDL-C, and iPTH, among other variables, were not significantly associated with ABI-defined PAD in any of the penalized analyses, as their 95% CI included zero.

Spearman correlation analyses were conducted to evaluate the relationships between ABI values, PCS levels, and clinical variables (Table 4). Following logarithmic transformation of skewed variables, serum PCS levels showed an inverse correlation with both left (*r* = −0.339, *p* = 0.001) and right (*r* = −0.357, *p* < 0.001) ABI. Furthermore, eGFR was inversely correlated with serum PCS levels (*r* = −0.253, *p* = 0.017).

**Figure 1:**
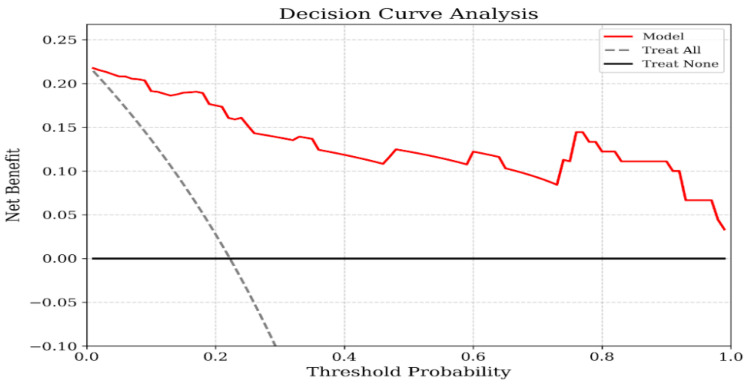
Decision curve analysis for the prediction model based on *p*-cresyl sulfate. The y-axis measures the net benefit. The x-axis displays the threshold probability. The red solid line represents the net benefit of using the *p*-cresyl sulfate-based model. The gray dashed line represents the assumption that all patients have the outcome (Treat All), while the black horizontal line represents the assumption that no patients have the outcome (Treat None). The decision curve shows that the model provides a higher net benefit across a wide range of threshold probabilities compared to the default strategies.

**Figure 2:**
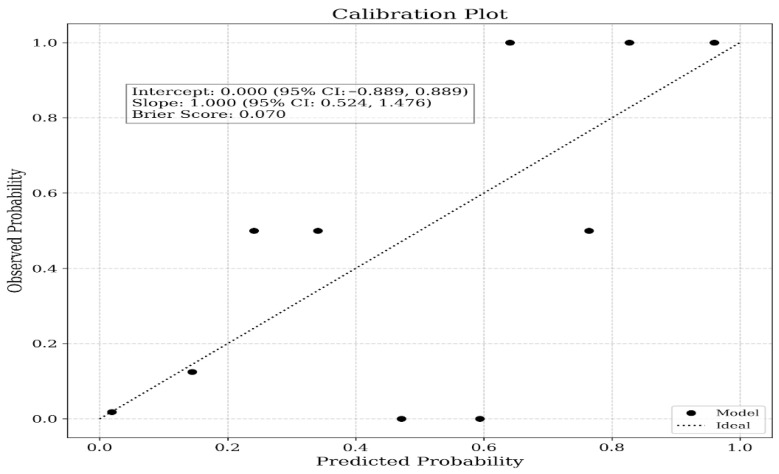
Calibration plot comparing predicted and observed probabilities of peripheral arterial disease across deciles of risk. The dashed line represents ideal calibration (perfect agreement between predicted and observed probabilities), while the points represent observed event rates across the model’s predicted probabilities (decile groups).

The Hosmer–Lemeshow goodness-of-fit test confirmed adequate calibration of the multivariate logistic regression model (χ^2^ = 4.130, *p* = 0.845). Decision curve analysis revealed that the PCS-based model provided greater net clinical benefit over a wider range of threshold probabilities than both the treat-all and treat-none strategies (Figure 1). Calibration metrics indicated reasonable agreement between predicted and observed risks, with an intercept of 0.000 (95% CI: −0.889 to 0.889) and a slope of 1.000 (95% CI: 0.524–1.476). The model also demonstrated good overall performance, as reflected by a Brier score of 0.070 (Figure 2).

## 4. Discussion

In this cohort of KT recipients, higher circulating PCS levels were associated with ABI-defined PAD. The association persisted after adjustment for conventional cardiovascular risk factors and renal function. PCS levels were also inversely correlated with ABI, indicating that higher toxin burden may be associated with more severe peripheral arterial involvement. Similar findings were observed in both multivariable and penalized regression models, suggesting a potential link between PCS levels and peripheral vascular impairment in KT recipients.

Diabetes mellitus is a well-established determinant of PAD and is among the strongest traditional cardiovascular risk factors in both the general population and transplant recipients [1,5,18,19,20]. Epidemiological data consistently demonstrate a substantially higher PAD prevalence among individuals with diabetes, with distal lower-extremity arteries particularly affected [20,21]. In our cohort, diabetes was more common in patients with ABI-defined PAD, although it did not remain significant after multivariate adjustment. The vascular consequences of chronic hyperglycemia are multifactorial. Sustained glucose exposure promotes endothelial dysfunction, oxidative stress, inflammatory activation, and prothrombotic changes, all of which contribute to arterial remodeling and reduced ABI values [20,22]. However, the inverse association between fasting glucose and ABI-defined PAD observed in the penalized models should be interpreted cautiously. In the conventional multivariable logistic regression analysis, fasting glucose showed only a borderline inverse association, and no clear correlation between glucose and ABI was observed in the Spearman analyses. Given the modest sample size and the limited number of PAD events, this finding may reflect model sensitivity, treatment-related influences on fasting glucose levels, overlap between diabetes status and contemporaneous glucose measurements, or residual confounding rather than a stable biological relationship. Accordingly, this result should be regarded as exploratory and hypothesis-generating rather than definitive. Notably, our study found no significant differences between the groups in other conventional risk factors, such as age, blood pressure, lipid profile, and renal function parameters. Even after accounting for diabetes and these traditional factors, PCS remained independently associated with ABI-defined PAD, underscoring its significance as an additional, nontraditional contributor to peripheral vascular impairment in KT recipients.

Prior studies have generally reported an inverse association between circulating PCS concentrations and renal function. In non-dialysis CKD populations, PCS accumulates progressively with declining kidney function and has been linked to adverse cardiovascular outcomes, including an increased risk of heart failure events, independently of eGFR [23,24]. In KT recipients, serum PCS levels were significantly higher in advanced CKD stages [12]. Ligabue et al. [8] further reported that PCS levels were influenced by prior vascular events and inversely associated with mature endothelial progenitor cell counts, suggesting that toxin accumulation after transplantation is influenced not only by graft function but also by recipient-specific factors. Consistent with these observations, we found that total PCS levels were inversely correlated with eGFR in our cohort. By contrast, the positive association between eGFR and ABI-defined PAD observed in the penalized models was not straightforward and should also be interpreted with caution. In the conventional multivariable logistic regression model, eGFR showed only a borderline positive association with PAD, and no significant correlation was observed between eGFR and either left or right ABI in the Spearman analyses. Given the modest sample size, limited event number, and the possibility that ABI-defined PAD in KT recipients reflects cumulative vascular injury that may not parallel contemporaneous graft function, this finding may represent statistical instability, model dependence, or residual confounding rather than a true protective or adverse effect of renal function itself. Taken together, these findings suggest that PCS and renal function may reflect related but distinct processes in vascular injury, and that PCS may provide additional information beyond eGFR regarding peripheral vascular involvement.

The present findings extend prior observations linking PCS to systemic vascular disease. Elevated PCS has been associated with arterial stiffness in patients undergoing hemodialysis [25], cardiovascular events in different clinical settings, including patients with ST-elevation myocardial infarction [26], and cardiovascular events and mortality in patients with CKD [27], implying that the vascular remodeling effects of PCS are generalized rather than confined to isolated vascular territories. Our study expands this evidence by demonstrating an association between PCS levels and ABI-defined PAD in KT recipients. PCS can induce oxidative stress and endothelial dysfunction by activating nicotinamide adenine dinucleotide phosphate (NADPH) oxidase signaling, increasing reactive oxygen species generation, and promoting vascular inflammation and smooth muscle cell remodeling. PCS has been implicated in vascular injury through oxidative stress, endothelial dysfunction, and vascular smooth muscle cell remodeling, which together contribute to arterial structural changes [15,28,29]. Animal and longitudinal human studies further support a role for PCS in atherogenesis and plaque progression [30,31]. Collectively, these data suggest that PCS functions not merely as a marker of vascular risk but also as an active mediator of endothelial injury and arterial remodeling. These biological mechanisms plausibly explain the association between PCS levels and ABI values observed in our cohort. Because PCS originates from intestinal microbial metabolism, interventions targeting gut-derived toxin production, such as dietary modification, prebiotics, probiotics, or oral adsorbents, have been proposed as potential adjunctive strategies [14,32]. Notably, synbiotic therapy has been reported to reduce plasma p-cresol levels in KT recipients [33]. Although these strategies were not evaluated in the present study, they highlight potential pathways for future research rather than immediate clinical application.

Given the cross-sectional design of this study, the directionality of the association between PCS levels and PAD cannot be determined. Several potential scenarios may explain the observed findings. First, elevated PCS levels may contribute to the development of PAD through mechanisms such as endothelial dysfunction, oxidative stress, and vascular remodeling. Alternatively, PAD may reflect an underlying burden of systemic vascular disease and impaired renal or metabolic function, which could also be associated with increased PCS accumulation. Finally, both PCS elevation and PAD may arise from shared underlying factors, including chronic inflammation, residual uremic toxin burden, or unmeasured clinical variables. These possibilities highlight the need for longitudinal studies to clarify the temporal and causal relationships between PCS and peripheral vascular disease.

Several limitations should be considered. First, given the cross-sectional and single-center design, a causal relationship between circulating PCS levels and ABI-defined PAD cannot be determined, and the temporal sequence remains unclear. Second, the relatively small sample size may have reduced statistical precision and may limit the generalizability of these findings, although consistent results were observed across different analytical methods. In particular, only 20 participants had ABI-defined PAD, whereas 9 covariates were included in the conventional multivariable logistic regression model. This limited events-per-variable ratio raises concerns regarding model stability, coefficient precision, and the possibility of overfitting. Although penalized regression methods were additionally applied to reduce overfitting and assess the robustness of the observed associations, these approaches cannot fully overcome the limitation imposed by the small event number. Therefore, the multivariable findings, especially those for covariates other than PCS, should be interpreted cautiously and regarded as exploratory. Third, only the total PCS was measured. As PCS is highly protein-bound, with the free fraction considered biologically active, total concentrations may not fully reflect biologically relevant exposure. This limitation may also restrict the mechanistic interpretation of the observed associations between PCS and vascular injury. Finally, residual confounding cannot be excluded. Important variables such as dialysis history, smoking status, lifestyle factors, gut microbiota composition, and other uremic solutes were not assessed. Larger, prospective, multicenter studies are needed to validate these findings.

## 5. Conclusions

Serum PCS levels were independently associated with ABI-defined PAD in KT recipients. Higher PCS concentrations were also correlated with lower ABI values, supporting a link between persistent uremic toxin burden and peripheral vascular impairment despite the restoration of renal function after transplantation. These findings support PCS as a nontraditional cardiovascular risk factor in this population. Further prospective studies are warranted to determine whether targeted modulation of PCS levels can improve vascular outcomes and reduce long-term cardiovascular risk in KT recipients.

## Figures and Tables

**Table 1:** Clinical variables of the 90 kidney transplantation patients in the normal or low ankle brachial index group.

Characteristic	All Participants (*n* = 90)	Normal ABI Group (*n* = 70)	Low ABI Group (*n* = 20)	*p* Value
Age (years)	54.39 ± 10.79	53.79 ± 10.92	56.50 ± 10.31	0.324
Post-KT duration (months)	94.17 ± 56.54	92.44 ± 55.20	100.20 ± 62.14	0.591
Dialysis duration (months)	71.40 (47.37–114.81)	71.40 (46.20–112.14)	76.68 (49.05–153.48)	0.415
Body mass index (kg/m^2^)	24.24 ± 4.42	24.07 ± 4.48	24.83 ± 4.28	0.502
Left ABI	1.04 ± 0.14	1.10 ± 0.09	0.86 ± 0.14	<0.001 *
Right ABI	1.05 ± 0.14	1.10 ± 0.09	0.88 ± 0.12	<0.001 *
Systolic blood pressure (mmHg)	141.30 ± 18.05	140.87 ± 17.40	142.80 ± 20.61	0.676
Diastolic blood pressure (mmHg)	84.42 ± 11.43	85.31 ± 11.43	81.30 ± 11.15	0.167
Total cholesterol (mg/dL)	187.47 ± 41.95	189.29 ± 43.92	181.10 ± 34.38	0.445
Triglyceride (mg/dL)	122.00 (86.75–173.25)	135.50 (88.75–181.50)	113.50 (81.50–132.75)	0.117
HDL-C (mg/dL)	50.00 (40.75–63.00)	50.00 (40.00–63.00)	50.00 (45.50–71.75)	0.475
LDL-C (mg/dL)	107.27 ± 35.37	109.86 ± 37.62	98.20 ± 24.64	0.195
Fasting glucose (mg/dL)	93.50 (87.00–112.50)	94.50 (88.75–119.00)	92.50 (79.25–97.50)	0.112
HbA1c (%)	6.30 (6.00–7.30)	6.20 (6.00–6.50)	6.40 (6.03–7.63)	0.285
Blood urea nitrogen (mg/dL)	25.00 (17.00–37.25)	25.50 (17.75–36.25)	19.00 (15.25–42.50)	0.341
Creatinine (mg/dL)	1.55 (1.18–2.10)	1.58 (1.23–2.10)	1.23 (0.89–2.10)	0.182
eGFR (mL/min)	46.94 ± 23.29	44.62 ± 20.54	55.07 ± 30.30	0.077
Total calcium (mg/dL)	9.37 ± 0.81	9.42 ± 0.83	9.19 ± 0.75	0.261
Phosphorus (mg/dL)	3.36 ± 0.82	3.33 ± 0.86	3.47 ± 0.68	0.510
iPTH (pg/mL)	88.85 (52.13–161.70)	106.45 (55.33–167.33)	68.40 (39.63–111.18)	0.113
Total *p*-cresyl sulfate (μg/mL)	17.26 ± 12.75	13.41 ± 9.86	30.71 ± 12.80	<0.001 *
Female *n* (%)	47 (52.2)	36 (51.4)	11 (55.0)	0.778
Diabetes *n* (%)	24 (26.7)	15 (21.4)	9 (45.0)	0.036 *
Hypertension *n* (%)	40 (44.4)	31 (44.3)	9 (45.0)	0.955
Living donor *n* (%)	13 (13.3)	9 (12.9)	3 (15.0)	0.804
Smoking *n* (%)	3 (3.3)	2 (2.9)	1 (5.0)	0.638
Tacrolimus use *n* (%)	65 (72.2)	50 (71.4)	15 (75.0)	0.753
Mycophenolate mofetil use *n* (%)	67 (74.4)	53 (75.7)	14 (70.0)	0.605
Steroid use *n* (%)	78 (86.7)	61 (87.1)	17 (85.0)	0.804
Rapamycin use *n* (%)	19 (21.1)	16 (22.9)	3 (15.0)	0.448
Cyclosporine use *n* (%)	18 (20.0)	14 (20.0)	4 (20.0)	1.000
Statin use *n* (%)	36 (40.0)	30 (42.9)	6 (30.0)	0.301
Fibrate use *n* (%)	16 (17.8)	13 (18.6)	3 (15.0)	0.713

Values for continuous variables are shown as mean ± standard deviation after analysis by Student’s *t*-test; variables not normally distributed are shown as median and interquartile range after analysis by the Mann–Whitney U test; values are presented as number (%) and analyzed by the chi-square test. ABI, ankle brachial index; KT, kidney transplantation; HDL-C, high-density lipoprotein cholesterol; LDL-C, low-density lipoprotein cholesterol; HbA1c, glycated hemoglobin; eGFR, estimated glomerular filtration rate; iPTH, intact parathyroid hormone. * *p* < 0.05 was considered statistically significant.

**Table 2:** Multivariable logistic regression analysis of the factors correlated to peripheral arterial disease among 90 kidney transplantation patients.

Variables	Odds Ratio	95% CI	*p* Value
*P*-cresyl sulfate, 1 μg/mL	1.254	1.108–1.419	<0.001 *
Diastolic blood pressure, 1 mmHg	0.931	0.851–1.019	0.121
Triglyceride, 1 mg/dL	1.007	0.997–1.018	0.178
LDL-C, 1 mg/dL	0.972	0.941–1.004	0.087
Diabetes, present	7.432	0.553–99.817	0.130
Fasting glucose, 1 mg/dL	0.963	0.926–1.002	0.061
Creatinine, 1 mg/dL	0.951	0.297–3.041	0.933
eGFR, 1 mL/min	1.059	0.991–1.131	0.090
iPTH, 1 pg/mL	0.998	0.991–1.025	0.609

The analysis data were obtained using multivariable logistic regression, with factors included if *p* < 0.2 (Table 1). LDL-C, low-density lipoprotein cholesterol; eGFR, estimated glomerular filtration rate; iPTH, intact parathyroid hormone. * *p* < 0.05 was considered statistically significant.

**Table 3:** Adjusted odds ratios and 95% confidence intervals for peripheral arterial disease derived from penalized logistic regression models.

Factors	LASSO β (95% CI)	LASSO *p* Value	Ridge β (95% CI)	Ridge *p* Value	Elastic Net β (95% CI)	Elastic Net *p* Value
*P*-cresyl sulfate, 1 μg /mL	2.63 (1.79, 4.32)	<0.001 *	1.39 (1.11, 1.69)	<0.001 *	1.40 (1.11, 1.71)	<0.001 *
Diastolic blood pressure, 1 mmHg	−0.90 (−1.68, 0.00)	0.146	−0.43 (−0.69, 0.06)	0.088	−0.41 (−0.69, 0.02)	0.112
Triglyceride, 1 mg/dL	0.91 (−1.60, 1.36)	0.716	0.53 (−0.58, 0.51)	0.898	0.51 (−0.56, 0.49)	1.000
LDL-C, 1 mg/dL	−0.64 (−2.50, 0.03)	0.160	−0.21 (−0.86, 0.05)	0.108	−0.19 (−0.86, 0.02)	0.128
Diabetes, present	0.18 (0.00, 1.84)	0.074	0.24 (0.08, 0.85)	0.024 *	0.22 (0.04, 0.84)	0.034 *
Fasting glucose, 1 mg/dL	−0.85 (−3.43, −0.17)	0.020 *	−0.50 (−0.94, −0.21)	0.002 *	−0.47 (−0.92, −0.16)	0.006 *
Creatinine, 1 mg/dL	0.13 (−1.28, 0.17)	1.000	0.12 (−0.45, 0.16)	1.000	0.08 (−0.42, 0.12)	1.000
eGFR, 1 mL/min	1.21 (0.09, 2.60)	0.034 *	0.65 (0.13, 0.95)	0.012 *	0.62 (0.12, 0.95)	0.012 *
iPTH, 1 pg/mL	−0.45 (−0.86, 0.97)	0.896	−0.35 (−0.52, 0.15)	0.236	−0.33 (−0.50, 0.11)	0.292

To validate the stability of the identified risk factors and mitigate potential overfitting due to the sample size, we performed penalized logistic regression analyses using LASSO, Ridge, and Elastic Net regularization. To estimate the uncertainty of the model coefficients, we utilized a bootstrap resampling procedure with 1000 iterations. The 95% CIs and empirical *p*-values for the odds ratios were derived from bootstrap distributions. LASSO, least absolute shrinkage and selection operator; β, standardized coefficients; CI, confidence interval; LDL-C, low-density lipoprotein cholesterol; eGFR, estimated glomerular filtration rate; iPTH, intact parathyroid hormone. * *p* < 0.05 was considered statistically significant (2-tailed).

**Table 4:** Spearman correlation coefficients between left ABI, right ABI, *p*-cresyl sulfate, and clinical variables in 90 kidney transplantation patients.

Variables	Left ABI		Right ABI		*P*-Cresyl Sulfate (μg/mL)	
	Spearman’s Correlation Coefficient	*p* Value	Spearman’s Correlation Coefficient	*p* Value	Spearman’s Correlation Coefficient	*p* Value
*P*-cresyl sulfate (μg /mL)	−0.339	0.001 *	−0.357	<0.001 *	—	—
Left ABI	—	—	0.674	<0.001 *	−0.339	0.001 *
Right ABI	0.674	<0.001 *	—	—	−0.357	<0.001 *
Age (years)	0.126	0.238	−0.025	0.818	0.006	0.957
Post-KT duration (months)	0.041	0.703	−0.031	0.775	−0.009	0.936
Log-Dialysis duration (months)	−0.176	0.097	−0.190	0.072	−0.028	0.794
Body mass index (kg/m^2^)	0.033	0.760	−0.034	0.747	0.013	0.900
SBP (mmHg)	0.161	0.130	−0.137	0.190	−0.199	0.060
DBP (mmHg)	0.001	0.991	0.059	0.581	−0.015	0.890
Total cholesterol (mg/dL)	0.001	0.991	0.065	0.540	0.010	0.927
Log-Triglyceride (mg/dL)	−0.031	0.772	0.149	0.160	0.071	0.504
Log-HDL-C (mg/dL)	−0.098	0.356	−0.114	0.285	−0.074	0.486
LDL-C (mg/dL)	−0.016	0.881	0.066	0.538	−0.029	0.785
Log-Glucose (mg/dL)	−0.022	0.834	0.052	0.624	0.003	0.975
Log-HbA1c (%)	−0.093	0.384	−0.129	0.225	0.113	0.290
eGFR (mL/min)	−0.132	0.216	−0.013	0.902	−0.253	0.017 *
Total calcium (mg/dL)	−0.106	0.319	0.196	0.064	0.073	0.496
Phosphorus (mg/dL)	0.168	0.112	−0.174	0.100	−0.082	0.443
Log-iPTH (pg/mL)	−0.071	0.503	0.139	0.192	0.202	0.056

Data on dialysis duration, triglycerides, HDL-C, glucose, HbA1c, and iPTH levels showed skewed distributions and were therefore log-transformed before analysis. ABI, ankle brachial index; KT, kidney transplantation; SBP, systolic blood pressure; DBP, diastolic blood pressure; HDL-C, high-density lipoprotein cholesterol; LDL-C, low-density lipoprotein cholesterol; HbA1c, glycated hemoglobin; eGFR, estimated glomerular filtration rate; iPTH, Intact parathyroid hormone. * *p* < 0.05 was considered statistically significant (2-tailed).

## Data Availability

Data supporting the findings of this study can be obtained from the corresponding author on reasonable request. Public data sharing is restricted because of ethical requirements and institutional regulations regarding participant confidentiality.

## Abbreviations

The following abbreviations are used in this manuscript:

ABI	Ankle–brachial index
BUN	Blood urea nitrogen
CKD	Chronic kidney disease
CI	Confidence interval
eGFR	Estimated glomerular filtration rate
HbA1c	Glycated hemoglobin
HDL-C	High-density lipoprotein cholesterol
iPTH	Intact parathyroid hormone
KT	Kidney transplant
LASSO	Least absolute shrinkage and selection operator
LDL-C	Low-density lipoprotein cholesterol
NADPH	Nicotinamide adenine dinucleotide phosphate
PAD	Peripheral artery disease
PCS	*P*-cresyl sulfate
TG	Triglycerides
